# Association Between Mycotoxin Exposure and Dietary Habits in Colorectal Cancer Development Among a Polish Population: A Study Protocol

**DOI:** 10.3390/ijerph17030698

**Published:** 2020-01-21

**Authors:** Katarzyna Eufemia Przybyłowicz, Tomasz Arłukowicz, Anna Danielewicz, Jakub Morze, Magdalena Gajęcka, Łukasz Zielonka, Bartosz Fotschki, Tomasz Sawicki

**Affiliations:** 1 Department of Human Nutrition, Faculty of Food Sciences, University of Warmia and Mazury in Olsztyn, Słoneczna 45F, 10-719 Olsztyn, Poland; katarzyna.przybylowicz@uwm.edu.pl (K.E.P.); anna.danielewicz@uwm.edu.pl (A.D.); jakub.morze@uwm.edu.pl (J.M.); 2 Department of Internal Medicine, School of Medicine, Collegium Medicum, University of Warmia and Mazury, 10-900 Olsztyn, Poland; tarlukowicz@wss.olsztyn.pl; 3 Department of Veterinary Prevention and Feed Hygiene, Faculty of Veterinary Medicine, University of Warmia and Mazury in Olsztyn, Oczapowskiego 13/29, 10-718 Olsztyn, Poland; mgaja@uwm.edu.pl (M.G.); lukasz.zielonka@uwm.edu.pl (Ł.Z.); 4 Institute of Animal Reproduction and Food Research, Polish Academy of Science, Tuwima 10, 10-748 Olsztyn, Poland; b.fotschki@pan.olsztyn.pl

**Keywords:** colorectal cancer, zearalenone, mycotoxin, dietary habits, study protocol

## Abstract

Colorectal cancer (CRC) is one of the most common and lethal types of cancer worldwide. The developing of this disease includes many factors such as genetic, socioeconomic, environmental, and lifestyle factors, and nutrition habits. The aim of the study is the determination of zearalenone and its metabolite level in the biological samples of participants at risk of CRC, in relation to the nutrition data and information on the quality of life dependent on health. In the cohort clinical trial, 150 participants aged between 50 and 65 will be studied. The participants will be assigned into two groups depending on the colonoscopy result. Participants will be tested at dietary intake, quality of life, sleep time and quality, stress level as well as biochemical parameters of the blood. Moreover, in the biological samples, concentration of zearalenone and its metabolites (α-zearalenol and β-zearalenol) as well as the characteristics of gastrointestinal bacterial will be determined, and the end of the trial for both groups and their results will be compared. Taking into account the possible effect of mycotoxins and nutrition habits on the development of cancer, the results obtained may allow the formulation of new nutritional recommendations and reduce the development and occurrence of CRC.

## 1. Introduction

Colorectal cancer is one of the most diagnosed cancers. It is the second most often detected cancer in females and the third in males [1,2,3]. The incidence of colorectal cancer (CRC) depends on the industrialization and high-developed of a given geographical region. The highest incidence rates are recorded in Western Europe, the United States, Canada, Australia, and Japan [2,4]. The reasons for the development of CRC are complex and related to genetic, socio-economic, environmental, and lifestyle factors as well as nutrition habits [5,6,7]. However, it is still unknown why the incidence of colorectal cancer is higher in the more developed countries [4]. At present, many screening assays (stool testing and colonoscopy) are being introduced in developed countries, which aim to reduce colorectal cancer incidence and mortality. Moreover, the most preventions programs recommend preventive examinations of asymptomatic adults over the age of 50 and are marked as an increased risk group [8,9]. 

Food safety is a major concern worldwide. In recent years, toxicity studies have assessed the effects of food contaminants such as mycotoxins in both in vitro and in vivo studies [10]. These secondary metabolites of fungi affect global agriculture so prolifically that they are virtually ubiquitous at a certain concentration in the human daily diet [11]. Mycotoxins are able to prevent degradation or degradation by juices and digestive enzymes present in the digestive tract of mammals [12]. These compounds also show stability in the digestive tract of ruminant farm animals, which allows mycotoxins to persist in meat and dairy products [12,13]. Moreover, thermal treatment such as cooking or freezing also does not break down some mycotoxins [14]. One of the groups of these compounds is zearalenone and its metabolites (α-zearalenol and β-zearalenol). Zearalenone is a non-steroidal estrogenic mycotoxin and is produced by several Fusarium species [12]. The exposure risk to humans and animals of this mycotoxin is the consumption of contaminated food and animal feeds [15]. Several studies show the effects of zearalenone and its metabolites of hormone-dependent tumors. A study conducted by Belhassen et al. [16] and Pillay et al. [17] noted a potential role of zearalenone and its derivatives in the risk of developing breast and cervical cancer. For these reasons, these compounds may affect the development of CDC. Furthermore, as mentioned above, diet is the dominant factor in human mycotoxin exposure, and it is suggested that the colon area is highly exposed to these compounds, and is, therefore, an important target for pathological development [18].

Therefore, the present clinical study will be conducted to investigate the correlations between lifestyle factors, nutrition habits, and mycotoxin levels in biological samples and colorectal cancer development among a Polish population.

## 2. Materials and Methods

### 2.1. The Aim of the Study

The study will be performed within the framework of the Polish Colonoscopy Screening Program. The program involves screening colonoscopies for the early detection of colorectal cancer in men and women aged between 50 and 65. The aim of the study is the determination of zearalenone and its metabolite (α and β) level in the blood plasma and feces of participants at increased risk of cancer, in relation to the nutrition data (FFQ) and information on the quality of life dependent on health (WHOQOL-BREF). The secondary aim is to examine correlations between gastrointestinal bacteria and colorectal cancer.

### 2.2. Ethical Aspects

The subjects will be informed about the potential benefits and risks of participation in the study. After this step, the participants will be asked to sign an informed consent form to participate in the study. The experimental design and procedures were approved by the Bioethics Committee of the Faculty of Medical Sciences of the University of Warmia and Mazury in Olsztyn (No: 6/2019). Moreover, the study was registered at http://www.clinicaltrials.gov (NCT04152265).

### 2.3. Characteristic of Participants and Study Design

**Figure 1:**
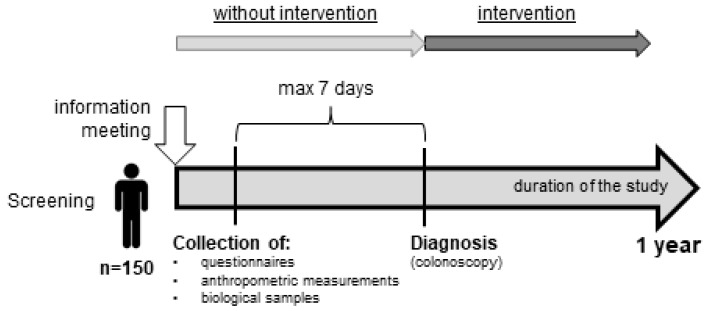
Time-based study design.

**Figure 2:**
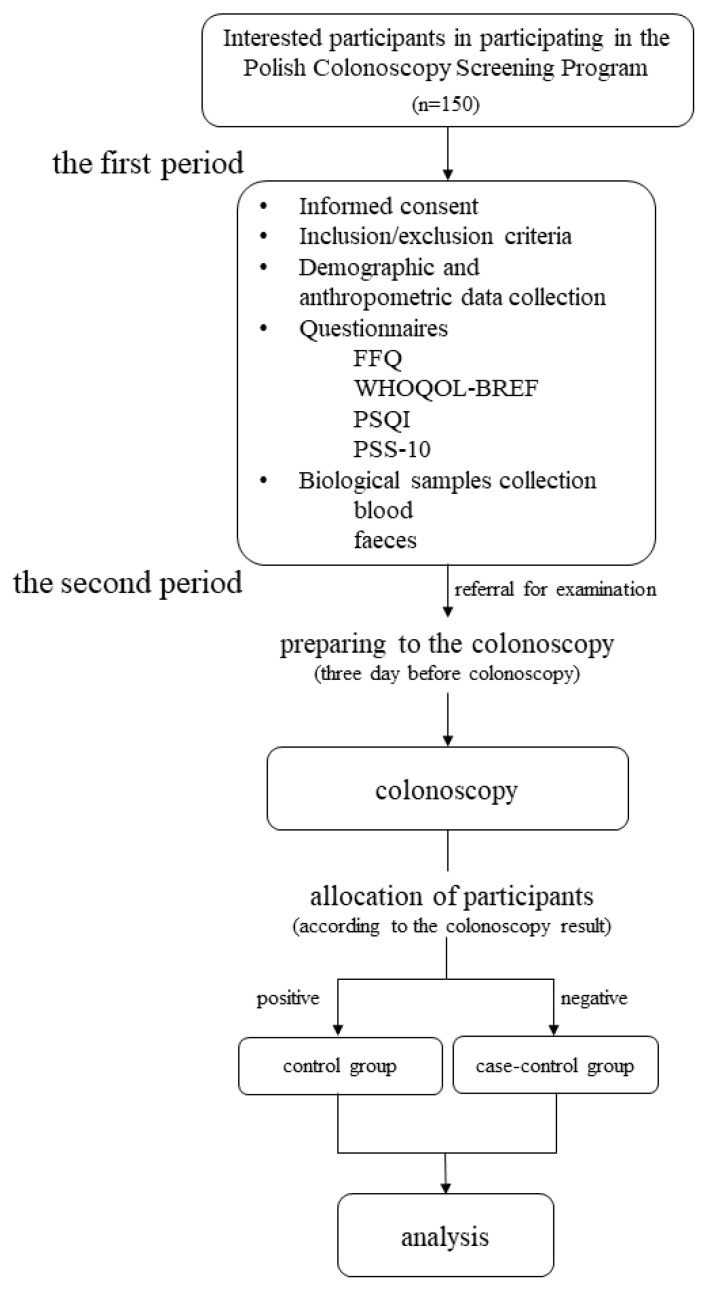
Protocol flow diagram. FFQ: Food frequency questionnaire; WHOQOL-BREF: World Health Organization Quality of Life Questionnaire; PSS-10: Perceived Stress Scale; PSQI: Pittsburgh Sleep Quality Index.

The study will be performed within the framework of the Polish Colonoscopy Screening Program. Men and women aged between 50 and 65 (*n* = 150) will take part in the study. This age group was chosen for the study because according to the literature adults over the age of 50 are marked as an increased risk group of CRC development [8,9]. The subjects will be recruited from among the patients of the Department of Gastroenterology of the Provincial Specialist Hospital in Olsztyn at least one month before the study. Within the study, 150 participants age between 50 and 65 will be invited to participate in the experiment. Participants will be excluded from the study if they are under 50 and over 65 years old, requiring long-term care due to somatic, mental retardation or other mental illness, or with a history of colorectal resection. The experiment will be divided into two periods (Figure 1). The first period (without intervention) will be one week before the second period (intervention—colonoscopy). During the first period participants will be invited to the Department of Human Nutrition, Faculty of Food Sciences, University of Warmia and Mazury in Olsztyn in order to collect the demographic and anthropometric data, collect the biological samples (blood and feces), and complete the questionnaires (food frequency questionnaire (German EEPIC FFQ2), the questionnaire of The World Health Organization Quality of Life (WHOQOL-BREF), The Pittsburgh Sleep Quality Index (PSQI), and the Perceived Stress Scale (PSS-10)). During the second period of the experiment, the participants will be referred for a colonoscopy. However, the endoscopic examination will take place no later than one week after the end of the first phase. The subjects will be asked to prepare for the colonoscopy, which involves cleansing the intestine from food leftovers. For this purpose, the preparation of Fortrans® will be used. Preparations for a colonoscopy also include diet restrictions such as three days before the colonoscopy, the participants should exclude from their daily diet baked products with grains, stone fruits (for example grapes, tomatoes, kiwi, strawberries), and linseed, poppy and sesame seeds; the day before the colonoscopy a light breakfast, cream soup for lunch, and liquids (water, tea, juices) will be recommended; and on the day of the examination only liquids are allowed (water, tea, juices), up to two hours before the scheduled colonoscopy. According to the colonoscopy results, the participants will be assigned to one of two groups. The schematic course of the experiment is shown in Figure 2.

### 2.4. Allocation of Participants

Participants, that will take in the screening colonoscopy, will be divided into two groups according to the result obtained. The first group will include the participants with a positive test result (control group), which will be characterized by the presence of polyps, adenomas, or neoplastic changes. While the second group (case-control group) will include the participants with negative test results. 

### 2.5. Demographic and Health Related Information

Demographic data will be obtained using the lifestyle and health questionnaire. Participants will be asked about age, sex, education level, employment status, place of residence, ethnicity, and living arrangement. Lifestyle information will include a declaration of physical activity level and time spent sitting (while TV-watching, reading, working with a computer). Questions about the overall quality of life (QOL) and sleeping time and quality will be assessed in separate questionnaires described below. Health-related information will be referred to as smoking status (current, former, passive, never) chronic diseases, non-communicable diseases, health history of the family, self-assessed health, and stress level.

### 2.6. Anthropometric Measurement

The nutritional status will be evaluated using anthropometric methods based on the following parameters: body weight measured by digital scale (SECA 515mBCA, Hamburg, Germany), height measured by stadiometer (Seca, Hamburg, Germany), waist and hip circumference measured by flexible nonelastic tape (Seca 203, Hamburg, Germany). These values will be used to calculate the following somatic indicators: body mass index (BMI; kg/m2), arm muscle circumference (AMC; cm), waist-hip ratio (WHR), and waist to height ratio (WtHR).

The analysis of body composition will be performed with the bioelectrical impedance method, using a Body Composition Analyzer SECA (515mBCA, Hamburg, Germany) with a weight limit of 300 kg and accuracy of 0.05 kg. This method consists of examining the intensity of a low electrical signal in 19 frequencies sent through the body through electrodes situated on the measurement platform. Body composition parameters determined with the use of the SECA include i.e., body mass (kg), body fat content (%), fat mass (kg), free fat mass (kg), muscle mass (kg), total body water (kg, %), extra- and intracellular water (%), visceral fat (in liters), basal metabolic rate (kcal), and segmental analysis of muscle mass, body fat, and their rating and phase angle (it correlates with metabolic status).

### 2.7. Sample Collection

The blood samples will be collected in the fasting state by a qualified nurse. Three blood samples will be taken from each participant. The first blood sample will be collected into a vacuum tube for the biochemical analysis, the second sample will be collected into a vacuum tube containing EDTA for hematologic analysis, while into the last tube the blood will be taken to the heparinized tube for plasma analysis. Directly, the first two tubes of blood samples will be transported on ice to the hospital laboratory, for blood parameters analysis (biochemical and hematological analysis). Whereas, the third tube with the blood will be centrifuged at 1000× *g* for 15 min at 4 °C, and the obtained blood plasma will be divided into aliquots, frozen, and stored at −80 °C until analysis. Besides, in this same day the fecal samples will be collected. The obtained samples will be separated into aliquots, frozen, and stored at −80 °C until analysis. 

#### 2.7.1. Analysis of Blood Parameters

The blood biochemical parameters of erythrocyte sedimentation rate (OB), amount of red blood cells (RBC), amount of white blood cells (WBC), hematocrit (HCT), mean corpuscular volume (MCV), lipid profile (total cholesterol, triglycerides, high-density lipoprotein (HDL), low-density lipoprotein (LDL)), creatinine, albumin, and level of glucose, adrenocorticotropic hormone (ACTH) and cortisol. The analysis of blood samples will be conducted by the Medical Diagnostic Laboratory of the Provincial Specialist Hospital in Olsztyn. Whereas, the concentration of zearalenone and its metabolites (α and β) in blood plasma samples will be assayed using the high-performance liquid chromatography coupled with mass spectrometry (HPLC-MS).

#### 2.7.2. Analysis of Fecal Samples

DNA of gut bacterial will be extracted with a commercial kit according to the manufacturer’s instructions. Samples will be mechanically lysed on FastPrep-24 on Zirconia beads and additionally enzymatically lysed towards bacteria. The presence of bacterial DNA in the samples will be confirmed using Real-Time PCR on a thermocycler with SYBR Green as fluorochrome. In the reaction for amplification of 16S rDNA the following universal reaction primers will be used: 1055F 5′-ATGGCTGTCGTCAGCT-3′ and 1392R 5′-ACGGGCGGTGTGTAC-3′. DNA will be quantified using the NanoDrop and standardized at 5 ng/µL. Microbial diversity will be studied by sequencing the amplified V3–V4 region of the 16S rRNA gene by using primers 16S. PCR conditions: 95 °C for 3 min; 25 cycles of: 95 °C for 30 s, 55 °C for 30 s, 72 °C for 30 s, 72 °C for 5 min, hold at 4 °C. The expected size on a Bioanalyzer trace after the Amplicon PCR step will be around 550 bp. Next, the amplicon pools will be prepared for sequencing and the size and quantity of the amplicon library will be assessed on NGS MiSeq. While, the obtained results will be combined with the amplicon library. 

The content and profile of short-chain fatty acids (SCFAs), which are indicative of the activity of gut microbiota, will be determined in the fecal samples using gas chromatography with a flame ionization detector (GC-FID).

The analysis of zearalenone and its metabolites in fecal samples will be determined by the method described in Section 2.7.1.

### 2.8. Dietary Assessment

Food intake will be evaluated based on validated self-administered, semiquantitative food frequency questionnaire (German EEPIC FFQ2) comprising 102 food items consuming within the last year. To calculate food and nutrient intakes predefined portion sizes are used. The FFQ was validated against two computer-assisted 24-hour dietary recalls. FFQ provides information about intake of 12 food groups and 18 nutrients [19].

### 2.9. Quality of Life Assessment

Quality of life (QOL) will be examined using a World Health Organization Quality of Life Questionnaire (WHOQOF-BREF). The WHOQOL-BREF instrument comprises 26 items, which measure the following broad domains: physical health, psychological health, social relationships, and environment. The WHOQOL-BREF is a shorter version of the original instrument that may be more convenient for use in large research studies or clinical trials [20].

### 2.10. Assessment Sleep Time and Quality

The Pittsburgh Sleep Quality Index (PSQI) is a self-administered questionnaire that assesses sleep quality within one month. It measures 19 individual items, generating seven component scores which consist of subjective sleep quality, sleep latency, sleep duration, habitual sleep efficiency, sleep disturbances, use of sleeping medication, and daytime dysfunction. The overall PSQI score ranges from 0 to 21, where higher score donates a poorer sleep quality [21].

### 2.11. Assessment of Stress Level 

The Perceived Stress Scale (PSS-10) is a brief and easy-to-administer measure of the perceived degree of one’s stressful life situations within the last month. It contains questions concerning various subjective feelings related to personal problems and events, behaviors, and methods of dealing with problems. The responses to the 10 items (including four items reverse-scored) are summing as psychological stress score and higher scores indicate greater psychological stress. PSS-10 has substantial reliability and validity and is a useful tool for examining the role of appraised stress levels in the etiology of disease and behavioral disorders [22,23].

### 2.12. Statistical Analysis

#### 2.12.1. Sample Size

Sample size calculation was based on the results published previously by Pillay et al. [17]. Due to the skewed distribution of data we recalculated median values and used an approach for log-normal outcomes proposed by O’Keffe et al. [24]. We determined that the sample size of 99 (48 per group) would achieve 80% power at the 5% significance level in order to detect a median difference of 0.1 μg/mL in plasma zearalenone between cancer and control groups. The available data (obtained from the Department of Gastroenterology of the Provincial Specialist Hospital in Olsztyn) provide that about 35% of colonoscopy results detect cancer changes. Thus, we decided to recruit 150 participants.

#### 2.12.2. Data Analysis

Data analyses will be performed using STATISTICA (version 13.1 PL, StatSoft Inc, Kraków, Poland), R software (version 3.5.2), and SPSS (version 25, IBM SPSS Statistics, New York, NY, USA). Normality of the variables will be examined using the Shapiro–Wilk test and, when needed, appropriate transformation (log or Box-Cox) will be applied. Continuous variables with normal distribution will be presented as means and standard deviations (SD) and Student’s *t*-test or one-way analysis of variance will be applied. Variables with non-parametric distribution will be presented as median and interquartile range and Mann–Whitney U test or Kruskal–Wallis test will be used. Categorical variables will be presented as a number and percentage and the chi-square test (χ^2^) or Fisher’s exact test will be used. The associations among variables will be evaluated using linear regression when the dependent variable is a continuous variable and logistic regression when the dependent variable is categorical. Potential confounders will be taken from the multivariable analyses, only if they show a weak association (*p*  <  0.20) against the dependent variables. The strength and direction of a correlation between two variables will be assessed using the Pearson correlation test. The level of statistical significance will be set at *p* < 0.05 for all tests. 

## 3. Discussion

Colorectal cancer is one of the common cancer diseases with an increasing prevalence throughout the world. The development of colorectal cancer is mainly asymptomatic and is detectable at the last stage of development [25], therefore examining relationships between various factors is one of the main aims of research. To the best of our knowledge, only scarce information regarding the level of zearalenone and its metabolites (α-zearalenol and β-zearalenol) and development of various type of cancer has been reported in literature so far [16,17]. The first study that evaluated the concentration of zearalenone and its derivatives in blood plasma to the correlation of breast and cervical cancer was presented. The obtained data suggested that presents of these compounds in blood does not indicate the unambiguous effect on the development of abovementioned types of cancer [17]. On the other hand, the authors noted that presence of zearalenone, α-zearalenol, and β-zearalenol in the blood samples can be used as an exposure indicator [17]. The second study focused only on the determination of mycotoxin (zearalenone and its metabolites) in urine samples and breast cancer risk [16]. The authors cited suggest a potential role of zearalenone in the risk of developing breast cancer, mostly one of the metabolites of zearalenone—α-zearalenol. The research cited above focused on other types of cancer than what we will study in our trial, but show the effect of the presence of mycotoxins in physiological fluids on the risk of cancer occurrence and development, which was very important and useful when creating this experiment.

In summary, it will be the first clinical trial to focus on the relations between mycotoxin exposure and dietary habits in colorectal cancer development. The results of this study may provide new information over the development of CRC and also have important nutrition implications for adults aged between 50 and 65 years old. 

## 4. Conclusions

This article described an original methodology to study the association between mycotoxin exposure and dietary habits in colorectal cancer development among polish population. The conducted research will allow broadening the knowledge about the correlation between the consumption of selected food groups and the risk of CRC in the aspect of mycotoxin consumption. Studies on the consumption of food groups as a potential source of mycotoxins and the increased risk of colorectal cancer have not been studied in Poland or worldwide. Therefore, the results obtained will form the basis of a methodology that can be used and adapted to study other types of cancer.
